# Effect of Interventions in WNT Signaling on Healing of Cardiac Injury: A Systematic Review

**DOI:** 10.3390/cells10020207

**Published:** 2021-01-21

**Authors:** Evangelos P. Daskalopoulos, W. Matthijs Blankesteijn

**Affiliations:** 1 Pôle de Recherche Cardiovasculaire (CARD), Institut de Recherche Expérimentale et Clinique (IREC), Université catholique de Louvain (UCLouvain), 1200 Brussels, Belgium; evangelos-panagiotis.daskalopoulos@uclouvain.be; 2 Department of Pharmacology & Toxicology, Cardiovascular Research Institute Maastricht, Maastricht University, 6229ER Maastricht, The Netherlands

**Keywords:** WNT signaling, myocardial infarction, infarct healing, in vivo, systematic review

## Abstract

The wound healing that follows myocardial infarction is a complex process involving multiple mechanisms, such as inflammation, angiogenesis and fibrosis. In the last two decades, the involvement of WNT signaling has been extensively studied and effects on virtually all aspects of this wound healing have been reported. However, as often is the case in a newly emerging field, inconsistent and sometimes even contradictory findings have been reported. The aim of this systematic review is to provide a comprehensive overview of studies in which the effect of interventions in WNT signaling were investigated in in vivo models of cardiac injury. To this end, we used different search engines to perform a systematic search of the literature using the key words “WNT and myocardial and infarction”. We categorized the interventions according to their place in the WNT signaling pathway (ligand, receptor, destruction complex or nuclear level). The most consistent improvements of the wound healing response were observed in studies in which the acylation of WNT proteins was inhibited by administering porcupine inhibitors, by inhibiting of the downstream glycogen synthase kinase-3β (GSK3β) and by intervening in the β-catenin-mediated gene transcription. Interestingly, in several of these studies, evidence was presented for activation of cardiomyocyte proliferation around the infarct area. These findings indicate that inhibition of WNT signaling can play a valuable role in the repair of cardiac injury, thereby improving cardiac function and preventing the development of heart failure.

## 1. Introduction

Myocardial infarction (MI) is one of the most frequent cardiovascular events and a major cause of heart failure (HF) development. Obstruction of the blood flow in coronary arteries results in a lack of oxygen and nutrients in the affected regions of the heart, causing the loss of cardiomyocytes (CMs) [1]. Despite major progress in the treatment of acute MI, achieved by developing technology to re-establish the flow through the affected coronary arteries, there is still damage inflicted to the heart in a significant fraction of the patients. This is due to reperfusion injury, insufficient success of the procedure (the ‘no-reflow’ phenomenon) [2] and late diagnosis of damage caused by plaque erosion, rather than plaque rupture [3].

In the infarcted heart, a wound healing response is initiated, resulting in the replacement of the injured CMs by scar tissue. Over the last decades, this wound healing response has been studied extensively. Following the death of CMs, an inflammatory response takes place first. This is followed by the formation of granulation tissue, rich in newly-formed blood vessels and extracellular matrix-producing cardiac fibroblasts (CFs). This granulation tissue eventually matures into scar tissue, which is characterized by large amounts of matrix with limited numbers of blood vessels and some myofibroblasts (MFs) [4]. Numerous studies have been published in which signaling pathways that can modulate the different processes involved in infarct healing, are described [1]. Moreover, there is increasing evidence that the damage can be repaired, at least in part, by inducing regeneration of CMs [5]. These studies can form the basis for the development of novel therapies that improve the infarct healing and diminish the development of HF.

One of the signaling pathways that has been extensively studied in the context of infarct healing is the WNT signaling pathway. After its initial discovery as a pathway involved in development and cancer, many other diseases and disease processes are now known to be regulated by WNT signaling [6]. Our group was the first to describe the upregulation of the expression of the seven transmembrane (7TM) receptor Frizzled-2 (FZD2) in cardiac hypertrophy [7] and MI [8]. In the meantime, a rapidly growing number of studies has been published in which WNT signaling was associated with many relevant processes in infarct healing, including CM apoptosis and regeneration, inflammation, angiogenesis and fibrosis [9].

The activation of WNT signaling during cardiac remodeling has been confirmed in many studies. An elegant tool to investigate this is the use of WNT signaling reporter mice. Using an axin-2 promoter-driven LacZ expression model, Oerlemans et al. [10] were the first to show activation of WNT signaling in endothelial cells (ECs), fibroblasts, leukocytes and Sca^+^ progenitor cells in the border zone of the infarct from 7 days post-MI onwards. Similar results were reported in other studies using axin-2 reporter mice [11,12], TOPGAL reporter mice [13,14] and a β-catenin-responsive construct of ferritin heavy chain and green fluorescent protein, carried by an adeno-associated virus serotype 9 (AAV9) [15]. In many of these studies, the activation of WNT signaling in the epicardium was reported during the initial phases of infarct healing, underscoring the relevance of this tissue in the orchestration of infarct healing [16,17]. It has to be noted, however, that all of these reporter models only show the activation of WNT-β-catenin signaling, leaving the potential role of non-β-catenin mediated (non-canonical) WNT signaling in the regulation of infarct healing underexposed (the reader can refer to Section 2 for an explanation of the different WNT signaling pathways).

The purpose of this systematic review is to provide a comprehensive overview of the studies on interventions in the WNT signaling in infarct healing. Because of the fact that the interplay between the various cell types involved in infarct healing is highly complex, we have decided to focus only on studies in which a direct intervention in WNT signaling was investigated in an in vivo model of MI (permanent coronary artery occlusion, ischemia/reperfusion (I/R) or cryoinjury). We have analyzed the effects of the interventions on critical parameters of infarct healing, including infarct size, cardiac function, inflammation, angiogenesis, fibrosis, and regeneration, in order to identify targets in the WNT signaling pathway with the most consistent beneficial effects.

## 2. WNT Signaling: An Increasingly Complex Network of Players and Pathways

WNT signaling was discovered more than 3 decades ago by simultaneous research on genes regulating pattern formation in Drosophila (wingless) and preferential integration sites for mouse mammary tumor virus (int1). A comparison of these studies’ results revealed that homologous genes were involved, resulting in the common name WNT for this gene family [18]. Since then, multiple components have been identified, making the signaling pathway an increasingly complex network of interacting extracellular and intracellular partners. Over the past years, many high-quality papers and reviews have been published on WNT signaling and its components. Therefore, we would like to refer the reader to these publications, included in the following paragraphs, for additional information. Here, we will restrict ourselves to a brief introduction of the components of the pathway that are targeted in the studies included in this systematic review.

Nineteen WNT proteins have been identified [19], which act as ligands for the receptors of the Frizzled (FZD) family [20]. Lipoprotein-related Receptor Protein (LRP) 5/6 and Ryk proteins have been found to act as co-receptors and to contribute to the signal transduction [21,22]. Members of the Dickkopf (DKK) family can serve as endogenous inhibitors of WNT signaling by interrupting the complex formation of FZD and LRP5 and -6, essential for the initiation of signaling [23]. In order to be biologically active, a palmitate group has to be attached to WNT proteins by an enzyme called Porcupine [24], and several synthetic inhibitors of this pathway have been developed that can be administered to reduce WNT secretion. An alternative way of interfering in WNT secretion is by deleting an essential chaperone protein called Wntless from the endoplasmic reticulum [25]. The activity of WNT signaling can also be regulated by proteins from the WNT inhibitory factor (WIF) and soluble frizzled-related protein (sFRP) families. These proteins can bind WNT in the extracellular space, preventing its interaction with the receptor complex and therefore inhibiting the signaling [26].

Different signal transduction pathways can be activated by WNT proteins. The most extensively studied is the WNT/β-catenin pathway, where β-catenin serves as the second messenger. This pathway is unusual in the sense that activation of WNT/FZD signaling inhibits the degradation of the second messenger, rather than stimulating its production. Under resting conditions, β-catenin is constantly degraded by a so-called “destruction complex”, consisting of axin, Adenomatous Polyposis Coli (APC) protein, glycogen synthase kinase-3β (GSK3β) and Casein Kinase-1 (CK1). This complex phosphorylates β-catenin, targeting it for degradation by the ubiquitin system. Upon activation of WNT signaling, however, this complex is disrupted and β-catenin is no longer phosphorylated, leading to its intracellular accumulation and migration to the nucleus, where it can interact with transcription factors from the T-cell factor/Lymphoid enhancer-binding factor (TCF/LEF) family to activate the expression of its target genes [27]. On the other hand, β-catenin-independent WNT signaling pathways have been described, including the WNT/planar cell polarity (PCP) [28,29] and WNT/Ca^2+^ pathways [30]. The PCP pathway is indispensable to transfer directional information during development. Defective PCP signaling is associated with malformations of cardiac outflow tract and spina bifida. Being an abundantly used second messenger, WNT-activated Ca^2+^ signaling can regulate multiple processes in the body. It has to be noted, however, that only few studies have addressed the role of β-catenin-independent WNT signaling in infarct healing so far.

## 3. Literature Search Method

**Figure 1:**
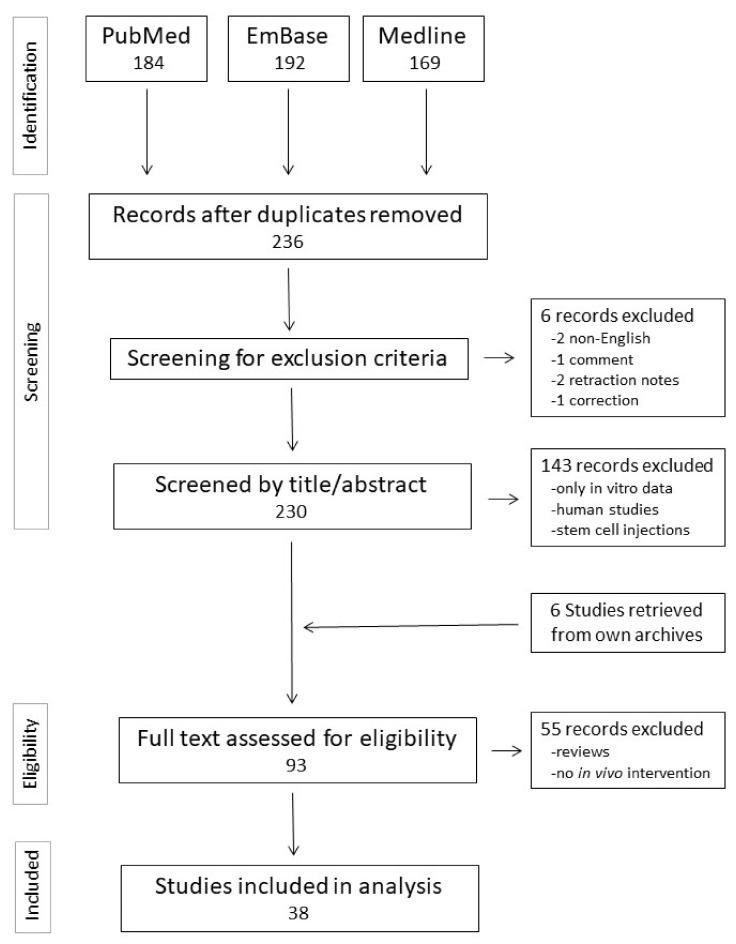
Flow chart of the selection of the articles, included in this systematic review.

Publications on WNT signaling and infarct healing were identified through the PubMed search engine (https://pubmed.ncbi.nlm.nih.gov/) using the search terms “WNT and myocardial and infarction”. The same combination of search terms was used in the EmBase and Medline search engines, assessed via Ovid at the Maastricht University Library. The final searches took place on 1 July 2020. After removing conference abstracts and reviews, the results were combined in a single database and duplicate publications were removed. Retraction notes, comments, and non-English publications were removed from the database. The results were analyzed according to the Preferred Reporting Items for Systematic Review and Meta-Analysis Protocols (PRISMA) [31]. A flow diagram of the search protocol is presented in Figure 1.

The abstracts from the publications in the database were assessed for eligibility by scanning for experimental data on interventions in WNT signaling in in vivo models of cardiac injury (permanent MI, I/R or cryoinjury). When the abstract was insufficiently clear, the full publication was evaluated. Manuscripts containing only in vitro data or biomarker studies in humans, review articles and editorials were excluded. This assessment for eligibility yielded 32 primary research articles. These were supplemented with 6 research articles from our own records that were not identified in the database searches but met the inclusion criteria, yielding a total of 38 research articles in this systematic review.

**Figure 2:**
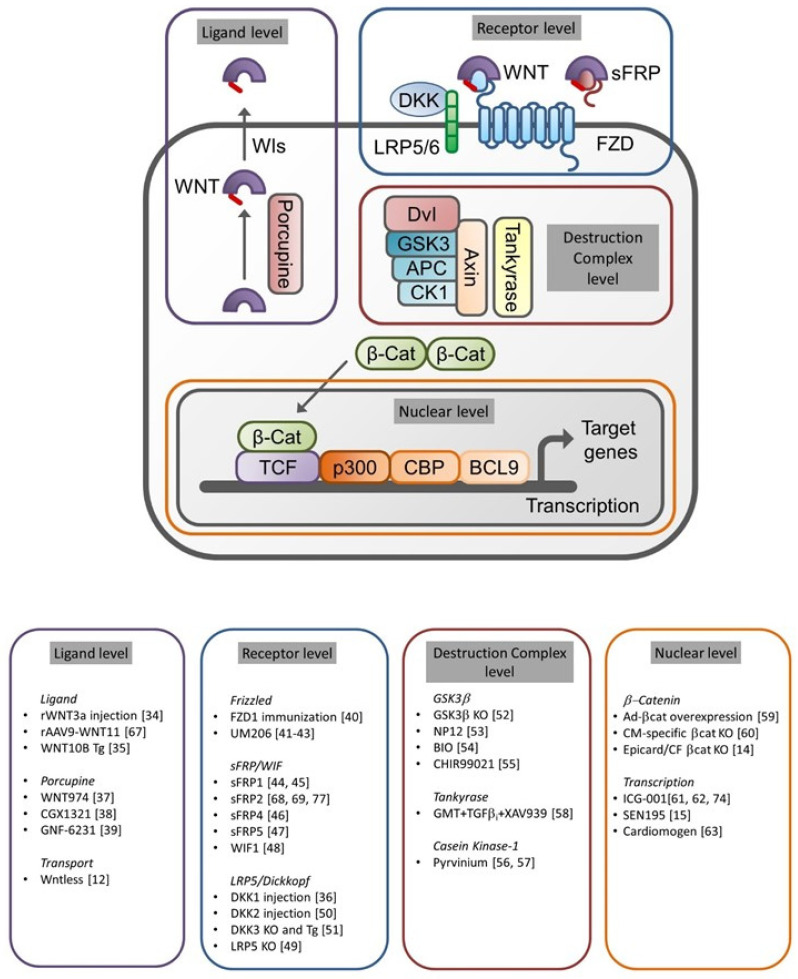
Schematic overview of the four levels in the WNT signaling pathway that we distinguished in our analysis: ligand level, receptor level, destruction complex level and nuclear level. In the lower part of the figure, the articles included in this systematic review are listed according to the level of intervention. Abbreviations: AAV9: Adeno associated virus serotype 9; Ad: adenovirus; βcat: β-catenin; DKK: Dickkopf; Dvl: dishevelled; FZD: frizzled; GSK3: glycogen synthase kinase-3; KO: knockout; LRP: LDL receptor-related protein; sFRP: secreted frizzled-related protein; WIF: WNT inhibitory factor; Wls: Wntless.

Full texts of the remaining manuscripts were analyzed for experimental data on the following topics: infarct size, cardiac function, CM apoptosis, inflammation, angiogenesis, fibrosis and CM proliferation. Moreover, we classified the interventions for each of the topics according to the level in the signal transduction cascade (ligand, receptor, destruction complex and nuclear), as illustrated in Figure 2. The results of this analysis are shown in Table 1. In the remainder of this systematic review, the results will be discussed following this classification.

## 4. Effect of Interventions in WNT Signaling on Infarct Size

As already mentioned earlier, myocardial damage is a well-established factor determining the infarct size following an acute ischemic event. The extent of the infarct size is directly associated with mortality [32] and therefore limiting infarct size is regarded as a critical strategy in the clinical setting, as well as an attractive therapeutic approach to preserve pump function after MI [33].

### 4.1. Ligand Level

In several studies, the WNT signaling pathway was targeted at the level of WNT production or WNT proteins were directly administered, either via injection or by overexpression. Oikonomopoulos et al. studied the effects of recombinant WNT3a (r-WNT3a) administered via injections in the border zone of the infarct area, immediately after the induction of permanent MI by occlusion of the Left Anterior Descending (LAD) coronary artery. The authors demonstrated that r-WNT3a injections led to a substantial enhancement in infarct size, a finding suggesting that activation of the WNT signaling pathway can be noxious for the infarcted heart, already 1 week post-MI [34]. On the other hand, another member of the WNT family, WNT10b, was genetically upregulated in a study by Paik et al. These authors utilized an αMHC-WNT10b transgenic (Tg) mouse overexpressing WNT10b specifically in the CMs and they demonstrated a ~2-fold reduction in infarct size in the Tg mice compared to wildtype (WT) mice, following myocardial cryoinjury [35]. In addition, the inhibition of the WNT-FZD-LRP interaction by insulin-like growth factor binding protein 4 (IGFBP-4), a LRP5/6 binding protein, was shown to confer a strong cardioprotective effect (infarct size suppression), compared to control mice. The authors provided in vitro evidence that IGFBP-4 affects WNT3a, but it cannot be excluded that other WNT family members might be affected by this protein [36]. These results indicate that the effect of direct administration of WNT protein on infarct healing can be strongly dependent on the isoform and mode of administration.

Porcupine inhibition is an attractive strategy to prevent acylation of WNT proteins, thereby suppressing their biological activity [25]. Moon et al. studied the effects of the porcupine inhibitor WNT-974 in a permanent (LAD) ligation mouse model. No direct infarct size data were reported, but it was shown that WNT-974 treatment might confer a modest reduction in scar area, 2.5 months following MI [37]. Another porcupine inhibitor, CGX1321, was administered for a shorter period (1 month) after permanent MI and was shown to substantially suppress infarct size (54% in control mice vs. 35% in their treated counterparts) [38]. Lastly, the porcupine inhibitor GNF-6231 was shown to halve the extent of infarct size within one month post-MI (control: 17% vs. GNF-6231-treated: 9%) [39]. Because porcupine inhibitors block the secretion and biological activation of WNT proteins in a non-selective fashion, these studies consistently show that a reduction in biologically active WNT proteins is beneficial for infarct size reduction. In contrast, macrophage-specific genetic deletion of Wntless, the crucial WNT ligand transporter, was not shown to confer any effects on the infarct size 1 month following permanent MI [12]. It would be, nevertheless, interesting to study whether a similar effect can be observed by deleting Wntless expression specifically in CMs.

### 4.2. Receptor Level

Interventions aiming to disrupt the interaction between ligand an receptor are a common strategy in the pharmacotherapy of cardiovascular diseases. Along these lines, antagonizing the interaction between the WNT ligand and its receptors would be a logical choice in an attempt to inhibit the WNT signaling pathway. This can be achieved by scavenging WNT proteins via a modulation of the levels of sFRPs or WNT Inhibitory Factor-1 (WIF-1), administering peptide fragments of WNT proteins or applying interventions to reduce the numbers of available receptors, as discussed below.

Injection of mice with mouse recombinant FZD-1 protein leads to an immune response against the FZD-1 receptor, attenuating the induction of FZD-1 expression in infarcted mice. Nevertheless, this approach did not confer any effect on infarct size, even though CM hypertrophy was attenuated in immunized mice [40]. Our group used a different approach in order to target the FZD receptors. We applied UM206, a peptide fragment of WNT3a/WNT5a proposed to block FZD-1 and -2, as a novel strategy to limit infarct size. Indeed, infarct size was substantially smaller when UM206 was administered via osmotic minipumps for 2 or 5 weeks after MI in Swiss mice [41]. This finding was confirmed in swine treated for 35 days with UM206 following I/R injury, as the infarct mass in UM206-treated pigs was strongly suppressed compared to control pig counterparts [42]. Nevertheless, in further studies elaborating on the mechanism of action of UM206, the beneficial effect on infarct healing could not be reproduced. The most likely explanation is that alterations in the experimental mouse model prevented the development of adverse remodeling, thereby eliminating the therapeutic basis for the beneficial effect of UM206 [43].

There is a relative abundance of studies focusing on the roles of proteins from the sFRP-family following cardiac ischemia. Genetic enhancement of sFRP1 expression was shown to reduce infarct size following LAD ligation or cryoinjury, either acutely (2 days post-injury) or chronically (15 or 30 days post-injury) [44]. A follow-up study with the transplantation of bone marrow cells from mice overexpressing sFRP1, showed that sFRP1 radically reduced infarct size. Nevertheless, this was not the case when sFRP1 expression was specifically enhanced in ECs or CMs, implying that the beneficial effect on infarct size is independent of the effects on the aforementioned cell types, but is rather mediated via inflammatory cells [45]. Matsushima et al. showed that intracardiac injection of sFRP4 after MI in rats suppressed infarct size, although no quantification was reported [46]. In the same direction, genetic deletion of sFRP5 led to a markedly increased infarct area compared to area-at-risk following I/R injury (WT: 29% vs. sFRP5 KO: 39%), indicating that sFRP5 is crucial for restricting the extent of the inflammatory response and thus, as a consequence, for limiting infarct size following I/R in mice [47]. On the other hand, another WNT ligand regulator, WIF-1, appears to play a crucial role in the magnitude of infarct size, by binding and neutralizing the WNT ligand, thereby turning WNT signaling off. Total KO of WIF-1 led to an exacerbation of infarct size (measured at 28 days post-MI), while the opposite effect was observed in mice overexpressing WIF-1 specifically in their CMs via AAV injection [48]. Overall, inhibition of WNT signaling by overexpression of sFRPs or WIF-1 appears to reduce infarct size in most of these studies.

Several studies addressed the effect of interventions in the co-receptor LRP5/6 and its endogenous inhibitors from the DKK family. LRP5 KO mice were found to demonstrate an aggravation in infarct size expansion compared to WT counterparts, whether this is expressed as a % of total left ventricular (LV) mass (WT: 10% vs. LRP5 KO: 21%) or as a % of the area-at-risk (WT: 17% vs. LRP5 KO: 30%) [49]. DKK2, a well-established inhibitor of the WNT/FZD signaling pathway that binds to LRP5/6, was shown to possess cardioprotective effects post-MI. DKK2 was injected in the infarct area and the border zone of rat hearts after I/R injury and infarct size was evaluated 1 week thereafter. The authors demonstrated an impressive 3-fold suppression of the infarct size in the DKK2-treated rats [50]. Bao et al. followed a more genetic–rather than a pharmacological–approach, to investigate the role of DKK3 following permanent MI [51]. The group utilized both DKK3 full KO and DKK3 CM-specific overexpressing mice and demonstrated–as expected–directly contrasting results. The magnitude of the infarct was substantially increased in the DKK3 KO mice compared to their WT counterparts (WT: 37% vs. DKK3 KO: 48%) but an opposite effect was observed in the DKK3 Tg (overexpressing) mice compared to their non-transgenic (NTG) littermates (NTG: 35% vs. DKK3 Tg: 23%). Taken together, both DKK2 and -3 appear to have a beneficial effect on infarct size, but this comes in contrast with the detrimental effect of LRP5 deficiency. These observations leave the role of WNT/LRP signaling in infarct healing unclear.

### 4.3. Destruction Complex Level

Our search identified seven studies where the effect of targeting the destruction complex on infarct size was investigated. Woulfe et al. utilized an inducible KO model where GSK3β was deleted specifically in CMs [52] and demonstrated a comparable scar length between the KO and the WT mice, indicating–indirectly–that the infarct size was similar between the two genotypes. Interesting work has been conducted in the last years with pharmacological inhibition of GSK3β, shedding light onto this important kinase as a regulator of infarct size. Baruah et al. investigated the potential effect of GSK3β inhibition on infarct size, after I/R (20 min of ischemia, followed by reperfusion for 7 and 14 days), by the agent NP12. The subsequent activation of the WNT/β-catenin signaling pathway following inhibition of GSK3β, was shown to attenuate infarct size, since the fibrotic area was approximately halved compared to controls at 7 days post-MI; at 14 days, the effect was still present but less prominent [53]. Similar results were presented by Kim et al., using the GSK3 inhibitor BIO (6-bromoindirubin-3′-oxime). Using a rat model of MI, they reported a more than 50% reduction of the scar size in the infarct area [54]. Although the results are not entirely consistent, it can be concluded that GSK3β inhibition tends to reduce infarct size in most of the studies. Finally, Badimon et al. studied the used a pharmacological approach in order to study the acute effects of GSK3β inhibition with SB415286 and demonstrated a robust reduction in myocardial damage in a mouse model of 60 min ischemia/90 min reperfusion, but no effect following ischemia without reperfusion. Nevertheless, the authors could not demonstrate a significant increase in β-catenin in their model, questioning the precise role of GSK3β in this protective response [55].

Focusing on non-GSK3β destruction complex targets has also been suggested as an alternative strategy to manipulate the metabolism of β-catenin. Pyrvinium, an agent that was found to prevent the degradation of axin, enhance CK1α and stabilize β-catenin (thus inhibiting WNT signaling) was used by Saraswati et al. following MI in mice. No effect was demonstrated on infarct size of a single intracardiac injection of pyrvinium compared to controls at 30 days post-MI [56]. In agreement with these findings, Murakoshi et al. demonstrated no effect on necrotic area in mice treated with pyrvinium by oral gavage from day 1–14 post-MI [57]. A slightly different strategy was followed by Mohamed et al. [58]. The authors compared the effects of a cocktail containing a TGFβ inhibitor (SB431542), a tankyrase inhibitor (XAV939–an axin stabilizing agent, blocking the accumulation of β-catenin and thus inhibiting the WNT signaling pathway) and GMT (the Gata4, Mef2c and Tbx5 cardiac transcription factors) in mediating reprogramming of CFs into CMs following MI. They indeed observed that 12 weeks post-MI, the aforementioned cocktail led to a strongly suppressed infarct size. Taken together, increasing the amount of β-catenin by inhibition of GSK3β appears to offer a consistent reduction of infarct size, whereas targeting other members of the destruction complex yields much more variable results.

### 4.4. Nuclear Level

The available literature focusing on the role of the WNT/β-catenin signaling pathway in cardioprotection at the nuclear level (i.e., relating to manipulations of β-catenin and its transcription factors) is strongly in favor of suppressing the signaling pathway, with the exception of a study by Hahn et al. and one by Zelarayán et al. In the first study, adenoviral gene transfer of β-catenin in a rat MI model attenuated infarct size by ~25% compared to the control rats. The authors proposed anti-apoptotic and pro-angiogenic mechanisms for this reduction in infarct size [59]. Zelarayán et al. investigated the effect of stabilization of β-catenin in the infarct size magnitude, however, no differences were observed in mice exhibiting β-catenin stabilization vs. control mice, 4 weeks post-MI [60].

In contrast, several studies (genetic and pharmacological) have been identified investigating the role of the inhibition of WNT/β-catenin signaling in infarct size magnitude. In this context, the same group [60] also used a constitutive CM-specific β-catenin KO mouse model and showed a substantial reduction in infarct size, 4 weeks after MI. They proposed a mechanism involving increased differentiation of resident cardiac progenitor cells into CMs to explain these results. Duan et al. used a Wilms tumor-1 specific β-catenin KO mouse, with a deletion specific in epicardial cells and a CF-specific β-catenin KO mouse. However, neither of the two genotypes demonstrated any difference in infarct size, compared to corresponding controls [14]. Furthermore, three groups investigated the effect of the WNT inhibitor ICG-001 on infarct size by blocking β-catenin/CBP interaction in various protocols. Sasaki et al. [61] did not observe any differences in infarct size in the ICG-001-treated rat hearts vs. controls, 4 weeks post-MI. In contrast, Sun et al. reported a substantial decrease in the infarct area of rats following ICG-001 treatment at 3 weeks post-MI [62], however using a considerably lower dose, via a different route and for a longer duration (hence, it is quite challenging to compare the two studies). Lastly, two further studies focused on inhibiting the signaling pathway at a TCF/LEF-mediated transcription level. The compound Cardiomogen 1 (CDMG1) was suggested by preliminary work to inhibit β-catenin and to negatively affect TCF/LEF-mediated transcription. Thus, CDMG1 was tested in a mouse permanent LAD ligation model and it exhibited a strong inhibitory effect on the scar length, indicating a smaller infarct [63]. In the same direction, Matteucci et al. applied the small molecule WNT inhibitor SEN195 in rats overexpressing a β-catenin responsive TCF/LEF reporter via AAV9 gene therapy. They demonstrated an impressive halving of the infarct size area, manifesting–as the vast majority of the aforementioned studies–that the inhibition of WNT/β-catenin signaling pathway is strongly associated with cardioprotective effects following MI [15].

## 5. Effects of Interventions in WNT Signaling on Cardiac Function

Following ischemic injury, the assessment of cardiac function (systolic and diastolic) is an essential step in monitoring the pathophysiologic changes taking place in the LV, the response to any therapeutic treatment and the overall clinical management of the subject. In the majority of cases in animal research, this is routinely performed by echocardiography [64]. Other methods that are frequently used to assess cardiac function are magnetic resonance imaging (MRI) [65] and measurement of LV pressure changes, by using a micromanometer [66]. Cardiac function was assessed in most of the studies included in this systematic review, as discussed below.

### 5.1. Ligand Level

Activation of the WNT signaling pathway via injections of r-WNT3a in the border zone had profound negative effects on cardiac function, as demonstrated by robustly reduced fractional shortening (FS) and fractional area change, when compared to vehicle-treated mice. Diastolic interventricular septum (IVS) thickness was also shown to be smaller in the r-WNT3a treatment group, but no difference was observed in the LV internal diameter in diastole (LVIDd) [34]. In a study utilizing a totally different strategy, WNT11 administration via rAAV9 was shown to confer cardioprotective effects at 2, 4 and 8 weeks following MI in mice. This was attested by a significant increase of ejection fraction (EF), cardiac output (CO) and stroke volume (SV) in the WNT11-treated animals. Nevertheless, WNT11 treatment did not result in any observable changes in end-diastolic volume (EDV) or end-systolic volume (ESV), compared to controls. Interestingly, the WNT11 administration via rAAV9 was performed 1 week before the MI induction, which makes the extrapolation to a clinical application rather challenging [67]. CM-specific overexpression of WNT10b did not affect cardiac function or architecture at baseline, although beneficial effects on relative changes in FS and LV internal diameter in systole (LVIDs)–indicating a protective effect on systolic function–were reported in a mouse model of cryoinjury [35].

Porcupine inhibition by administration of WNT-974 for 10 weeks following permanent MI in mice led to a substantial increase in cardiac function (an improvement of EF from 25% in controls to 38% in the treatment group, as shown by MRI data) and a suppression of LV dilatation (as attested by a lower ESV in the WNT-974 treated mice) [37], indicating a cardioprotective effect. Similarly, CGX1231 treatment was shown to improve EF and FS, while enhancing LV posterior wall (LVPW) thickness in diastole, suggesting not only a halting of LV dilatation, but also a protective effect on cardiac function [38]. Notably, GNF-6231 administration improved marginally (but significantly) both EF and FS and had beneficial effects on LVIDd and LVIDs, limiting dilatation of the LV at 30 days–but not as early as 7 days-after permanent ligation of the LAD [39]. Taken together, these results point towards a beneficial effect of Porcupine inhibition on cardiac function post-MI.

Macrophage-specific deficiency of Wntless results in substantially improved cardiac function (enhanced EF and FS) and preserved LV architecture (increased LVPWs thickness and suppressed LV end-systolic-area), 30 days post-MI [12].

### 5.2. Receptor Level

Fan et al. showed that an immunization strategy against the FZD-1 receptor can have a profound effect on FZD-1 mRNA and protein levels, and a drastic inhibition of active β-catenin levels. This was associated with effects on systolic (a slight improvement of EF, compared to controls) and hemodynamic function (enhancement of dP/dt max, dP/dt min, LVESP and a suppression of LVEDP). Interestingly, the LVIDd was reduced in the immunized mice, while LV anterior wall thickness in diastole was attenuated, signifying cardioprotective effects [40]. The administration of UM206 for 5 weeks following MI profoundly improved systolic function (by almost doubling the EF), prevented dilatation (by suppressing EDV by ~40% compared to control-treated mice) and greatly improved hemodynamic parameters (dP/dt max and min) [41], albeit these results could not be reproduced in later studies as discussed before [43]. The application of the same treatment to a swine model of I/R did not result in a significant improvement of pump function, but it has to be noted that the infarcts in this study were relatively small compared to those induced in mice, and therefore the pump function was only mildly impaired [42].

Barandon et al. showed that the genetic overexpression of sFRP1 can have a beneficial effect on cardiac function 2 weeks post-MI, as attested by improved dP/dt measurements (with or without dobutamine challenge) [44]. In a follow-up study, the same group demonstrated similar effects on dP/dt measurements in mice transplanted with bone marrow cells from Tg mice overexpressing sFRP1. Interestingly, genetic overexpression of sFRP1 specifically in ECs, produced similar results [45]. Unfortunately, neither of these two studies investigated any other parameter of cardiac function or architecture (e.g., via echocardiography). Inactivation of sFRP2 via a genetic deletion in mice also demonstrated a cardioprotective phenotype, with substantial improvements in cardiac function (EF) in sFRP2 null mice, 14 days after ischemia [68]. Interestingly, LV function 4 weeks post-MI was not affected compared to vehicle, when sFRP-2 levels were increased following injection of sFRP-2 in the infarct area in rats [69], while anterior wall thinning was halted compared to control rats. The injection of sFRP4 via different modes of administration could also improve cardiac function (enhancing EF at different time points) after MI in rats [46]. Lastly, sFRP5 null mice exhibited an exacerbated LV systolic dysfunction and dilatation, at 2 and 4 weeks post-I/R, indicating a cardioprotective role of sFRP5 following this type of cardiac injury [47]. Lastly, targeting factors affecting WNT ligand transport have also been shown to confer important effects on cardiac function and remodeling. The total genetic deletion of WIF-1 was reported to have devastating effects on systolic function, one month following permanent LAD ligation. Inversely, the CM-specific overexpression of WIF-1 (via AAV9) was shown to substantially improve EF and FS (both were more than doubled compared to AAV-control mice) [48].

The therapy with the WNT-FZD-LRP interaction inhibitor IGFBP-4/H95P (H95P preventing from binding with the insulin-like growth factor but still allowing WNT inhibition) conferred very impressive effects on cardiac function when injected in mouse infarcts. Mice injected with IGFBP-4/H95P showed improved cardiac function (enhanced EF and FS) from 2 weeks up to 20 weeks post-MI [36].

Knocking-out of LRP5/6 was not shown to improve cardiac function (as demonstrated by EF and FS) or architecture (LVIDd and LVIDs) in a mouse MI model, 2 weeks after infarction. In the same study, a single injection of the DKK1 protein shortly after MI did not result in any observable effects on cardiac function and architecture compared to control mice, 4 weeks after permanent MI [36]. On the contrary, a cardioprotective effect was conferred on infarcted rat hearts following DKK2 treatment, 3 weeks post-I/R. Indeed, DKK2-treated rats showed an impressive enhancement of EF (~29% increase) and FS (~22% increase) to the level of uninfarcted hearts [50]. Similar cardioprotective effects were demonstrated by a CM-specific genetic study, where overexpression of DKK3 in mice contributed to a substantial improvement in cardiac function (EF, FS, dP/dt max and dP/dt min, end-systolic and -diastolic pressures) and attenuation of dilatation (reduction of LVEDd and LDEDs). In contrast, a genetic deletion of DKK3 in mice led to a deteriorated cardiac function, as attested by worsened EF, FS, dP/dt max and dP/dt min and end-systolic pressure, as well as aggravated dilatation (with respective increases in LVEDd and LDEDs) [51]. These results suggest that different members of the DKK family have opposing effects on cardiac function in the infarcted heart.

### 5.3. Destruction Complex Level

Various groups have studied the effects of manipulations at the level of the destruction complex by affecting GSK3β or CK1. Conditional genetic deletion of GSK3β in CMs, was shown to confer cardioprotective effects following permanent MI in mice. Indeed, it was shown that GSK3β KO mice exhibit improved diastolic and systolic function post-MI, with EF improving already at 4 weeks post-MI, preserved LVEDD being evident after 6 weeks and hemodynamic parameters improving at 8 weeks post-MI [52]. Inhibition of GSK3β by the allosteric inhibitor NP12 led to better systolic (enhancement of EF and FS) and diastolic (as attested by clear effects on deceleration time) function compared to controls, 7 and 14 days post-MI in a mouse I/R model [53]. Major systolic function-related (EF, FS) and LV architecture-related (LVIDd, IVDs, LVIDs, ESV) echography parameters were found to be substantially improved, following daily treatment of rats with the GSK3β-inhibiting agent BIO, 14 days after permanent MI [54]. These results consistently show that activation of WNT/β-catenin signaling by inhibition of GSK3β has a beneficial effect on cardiac function.

The CK1α enhancer and axin stabilizer pyrvinium failed to demonstrate a beneficial effect on cardiac function when administered as a one-off intracardiac injection following MI [56]. The group of Murakoshi et al. used a different strategy, administering pyrvinium intragastrically from day 1- 14 post-MI. They demonstrate a significant enhancement of EF compared to controls at d7 and d14 post-MI, without reporting any other echocardiographic parameter or proving the inhibitory effects of pyrvinium on WNT signaling [57], rendering the interpretation of this result from a WNT-signaling point-of-view, puzzling. The use of an axin stabilizer, XAV939, as part of a treatment cocktail also entailing a TGFβ inhibitor (SB431542) and GMT, was shown to substantially improve cardiac function following MI in mice. Twelve weeks following MI injury, the mouse group receiving the aforementioned cocktail therapy exhibited a substantial improvement in systolic function, as reflected by a significant increase in EF, CO and SV, compared to controls [58].

### 5.4. Nuclear Level

In our systematic research, only two studies were identified in which the role of the overexpression of β-catenin on cardiac function was investigated. Hahn et al. studied female Sprague-Dawley rats following adenoviral gene transfer of β-catenin (Ad-β-catenin) or control (Ad-GFP) and showed that the upregulation of β-catenin expression led to an improvement of systolic function (enhanced FS) and halting of LV dilatation (reduced LVEDD), 7 days post-MI [59]. Additionally, CM-specific stabilization of β-catenin-leading to enhancement of the WNT/β-catenin signaling-failed to establish any quantifiable effects on cardiac function of mice, 1 month post-MI [60].

Τhe CM-specific genetic deletion of β-catenin improved systolic dysfunction (as shown by the enhancement in EF, FS and SV) compared to control mice, but shows no effect on the halting of dilatation, 2 weeks post-MI [36]. A similar Tg mouse (conditional CM-specific β-catenin deletion) exhibited similar effects on systolic function, as reported by Zelarayán [60]. Indeed, β-catenin KO mouse hearts demonstrated improved FS (an increase by ~6% in β-catenin KO mice) and thus cardiac function 4 weeks after MI, but yet again, no differences were observed in other echocardiographic parameters, like IVSd or LV dimensions. On the other hand, the epicardial cell-specific or CF-specific deletion of β-catenin revealed a different picture. Deletion of β-catenin in both cell types, was associated with worsened FS and increases in EDV/ESV (implying exacerbated dilatation) 8 days post-I/R, compared to control mice expressing normal β-catenin [14]. The pharmacological inhibition of WNT/β-catenin signaling by ICG-001 led to an improvement of systolic function in two rat MI models. Indeed, Sasaki et al. showed a small–but significant–improvement in EF following a 10-day subcutaneous ICG-001 administration at 4 weeks post-MI in female rats [61], although there was no difference in parameters like LV thickness or LV volume. Another study by Sun et al. reported similar results with a clear improvement of EF and FS in the ICG-001-treated rats and important differences in the internal dimensions of the LV, indicating that ICG-001 is attenuating dilatation [62]. Lastly, beneficial effects on cardiac function are also shown by compounds inhibiting the WNT signaling pathway at the Tcf/Lef transcription level. Administration of the small molecule β-catenin/TCF inhibitor SEN195 exhibited a strong ~1.5-fold increase in EF and important reductions in LV volumes in both systole and diastole, in a mouse MI model [15]. Notably, similar beneficial effects in EF and FS and LVIDD were conferred by CDMG1 i/p injections when administered for 27 days in mice following MI [63]. It is clear that the conclusions from the above studies point to an attenuating effect of WNT/β-catenin signaling inhibition on cardiac dysfunction and thus, a beneficial effect in attenuating LV remodeling.

## 6. Effect of Interventions in WNT Signaling on Cardiomyocyte Apoptosis

A characteristic feature of MI is the loss of cells, both CMs and non-CMs, due to the ischemia in the infarct area. Cells can die either in a non-regulated (necrosis) or regulated (e.g., apoptosis) fashion. Extensive research has shown that the cell loss due to permanent ischemia is mainly non-regulated, whereas regulated cell death typically occurs as a result of reperfusion. In the past decades, multiple mechanisms of regulated cell death have been identified [70]. In the literature that was analyzed in this systematic review, however, we only found reports on apoptosis so we focus on this form of regulated cell death in the following part.

### 6.1. Ligand Level

Two studies have been published in which the effect of administration of a porcupine inhibitor on CM apoptosis was investigated in vivo, using the Terminal deoxynucleotidyl transferase dUTP nick end labeling (TUNEL) technique. In both studies, cardiac ischemia was induced by permanent ligation of a coronary artery, which has been reported to induce cell necrosis rather than apoptosis [70]. Despite this technical shortcoming, Bastakoty et al. reported a 2-fold reduction in the number of TUNEL^+^/cTnI^+^ CMs in mice treated with GNF-6231 [39]. In contrast, the number of TUNEL^+^ CMs was unaltered by the administration of CXG-1321, although a trend towards a reduction could be observed [38]. These observations suggest that inhibition of WNT secretion by porcupine inhibitors results in a tendency to reduce CM apoptosis in the ischemic areas of the heart post-MI, although confirmation in a model showing more CM apoptosis, such as I/R, would be required to draw firm conclusions. Overexpression of WNT11 via AAV9-mediated gene transfer did not affect the number of apoptotic cells at 24 h post-MI [67].

### 6.2. Receptor Level

The effect of interventions at the receptor level on CM apoptosis was investigated in six studies. Immunization of mice with recombinant FZD1 protein did not result in a change in relative myocardial apoptosis at 7 days post-MI [40]. Using local injection of DKK2 protein around the infarct, Min et al. reported a ~>3-fold reduction of the apoptotic index in an I/R model of cardiac ischemia [50]. Bao et al. compared the effect of overexpression and knockout of DKK3 and observed an increase of TUNEL^+^ cells in the peri-infarct zone in the knockout and a decrease in the overexpressing mice, upon permanent LAD ligation [51]. Overexpression of the sFRP1 analogue FrzA resulted in a ~2-fold reduction in TUNEL^+^ cells in the infarct area at 7 days after permanent LAD ligation; the apoptotic index was decreased by 50% [44]. In contrast, direct injection of sFRP2 around the infarct area did not affect the numbers of apoptotic cells in the border zone [69]. Finally, a doubling of the number of TUNEL^+^ cells was reported in sFRP5-knockout mice, 24 h after I/R [47]. These studies show that the presence of inhibitors of WNT signaling reduces the number of TUNEL^+^ cells in the infarct area, albeit in none of the studies the apoptotic cells’ identity was identified, e.g., by performing additional immunohistochemical characterization.

### 6.3. Destruction Complex Level

Apoptosis of cells in/around the infarct area was assessed in two of the seven studies where interventions in the destruction complex were applied. CM-specific deletion of GSK3β was found to substantially reduce the number of apoptotic CMs in the remote myocardium at 8 weeks post-MI [52]. On the other hand, a single intracardiac injection of the CK1-inhibitor pyrvinium did not result in a significant change in the number of caspase-3 positive cells at 30 days post-MI [56]. Given the small number of studies, the effect of interventions at the level of the destruction complex on apoptosis remain inconclusive.

### 6.4. Nuclear Level

The effect of interventions at the nuclear level on apoptosis were reported in three of the studies. Hahn et al. showed a ~30% reduction in TUNEL^+^ cells in AAV9-β-catenin injected to infarcted rat hearts at 7 days post-MI. Using conditional CM-specific depletion of β-catenin, Zelarayán et al. did not find evidence for an alteration in the rate of CM apoptosis at 4 weeks after permanent LAD ligation [60]. In contrast, at 21 days after permanent LAD ligation in rats, administration of ICG-001 resulted in a reduction in the number of apoptotic cells. Investigation of the levels of apoptosis-related proteins revealed a reduction of cleaved caspase-3 levels and an increase in the Bcl-2/Bax expression level, indicative of an anti-apoptotic effect of the ICG-001 treatment [62]. However, since the cell type(s) staining positive with TUNEL were not identified, the studies are inconclusive regarding the effect of the interventions on apoptosis in CMs.

## 7. Effect of Interventions in WNT Signaling on Inflammation

Within hours after MI, an acute inflammatory response is initiated, consisting of the infiltration of the infarct area with leukocytes. The first wave of inflammatory cells is formed by polymorphonuclear neutrophils (PMNs), followed by a wave of macrophage infiltration, peaking at ~7 days post-MI [1]. This inflammatory response is of vital importance for the removal of necrotic debris and extracellular matrix, making way for the cells that form the granulation tissue to enter the infarct area [71]. However, excessive inflammation can affect the integrity of the ventricular wall and lead to infarct rupture [72], underscoring the importance of a tight control of the inflammatory response. Many signaling molecules and pathways are known to modulate the inflammatory response after MI, including WNT signaling [73]. An overview of the studies in which the effect of interventions in WNT signaling on the inflammatory response after MI is reported is provided below.

### 7.1. Ligand Level

Overexpression of WNT11 via AAV 9-mediated transduction in a mouse model of MI with GFP-labeled bone marrow cells resulted in a more than 2-fold reduction in bone marrow-derived cells in the border zone at 4 and 8 weeks post-MI, and even stronger reductions in the non-infarct area. Additional experiments showed reduced presence of CD45^+^ (leukocyte common antigen) and CD68^+^ (macrophage antigen) inflammatory cells in the non-infarct region at 1 week post-MI in the AAV9-WNT11 treated group. This was accompanied by a marked reduction in the expression of multiple inflammatory cytokines [67]. CM-specific overexpression of WNT10b also resulted in reduced CD45^+^ immune cells and CD11b^+^/Ly6G^+^ immune cells at 5 days after cryoinjury [35]; here it has to be noted that in contrast to WNT11, WNT10b is considered to activate β-catenin-mediated WNT signaling. In the study of Palevsky et al., it was shown that macrophages in the infarct area secrete WNT proteins that stimulate the non-β-catenin mediated WNT signaling. Interruption of this secretion by knocking out the Wntless gene in myeloid cells changed the macrophage phenotype to the anti-inflammatory M2 subtype with reparative and pro-angiogenic properties. Although the inactivation of Wntless will affect the secretion of all WNTs, and not just WNT11, the results from the latter study seem to contradict those from the WNT11 overexpression experiments [12].

### 7.2. Receptor Level

Interventions at the level of WNT receptors and extracellular scavengers were also investigated in the context of inflammation. In the FrzA overexpressing mouse model, the number of myeloperoxidase-positive cells (mainly PMNs) was found to be significantly lower at day 2 and day 7 post-MI, indicating an attenuated first wave of inflammation. Macrophage and lymphocyte counts, on the other hand, were not different between Tg and control mice, suggesting a normal second wave of inflammation [44]. Building on this observation, researchers from the same group transplanted sFRP1 Tg bone marrow cells in WT mice and could recapitulate the reduced neutrophil infiltration in the infarct area at 2 and 7 days post-MI. They concluded that sFRP1 suppressed the neutrophil activation by inhibiting the production of the relevant cytokines [45]. Along similar lines, interventions in WIF-1 were shown to affect the ratio of inflammatory (Ly6C^high^) to resident (Ly6C^low^) monocytes: in WIF-1 knockout mice, a significant increase in inflammatory monocytes was observed, whereas AAV9-mediated overexpression of WIF-1 attenuated the monocyte response at day 4 post-MI [48]

In a study where the effects of overexpression and knockdown of DKK3 were compared in a mouse MI model, DKK3 was found to have an anti-inflammatory effect, 1 week post-MI, whereas an increased inflammatory response was observed in the DKK3^-/-^ mice [51]. A similar result was obtained in a study where infarct healing was investigated in mice lacking sFRP5, challenged by I/R injury. The sFRP5^-/-^ mice showed more infiltration of WNT5a-positive macrophages and higher levels of pro-inflammatory cytokines TNFα, IL-1β and MCP-1 compared to WT [47].

### 7.3. Destruction Complex Level

The effect of interventions at the level of the destruction complex on inflammation were addressed in a single study. Administration of the allosteric GSK3β inhibitor BIO shifted the macrophages in the infarct area from the M1 to the anti-inflammatory M2 phenotype at 14 days post-MI, suggesting that stimulation of WNT-β-catenin signaling at the level of the destruction complex has an anti-inflammatory effect [54].

### 7.4. Nuclear Level

Effects on inflammation were not reported in any of the studies in which interventions at the nuclear level of WNT/β-catenin signaling were investigated.

## 8. Effect of Interventions in WNT Signaling on Angiogenesis

The formation of new blood vessels in the area of infarction is essential for successful repair, as the cells that are required in the repair process need an adequate supply of oxygen and nutrients. Early signs of neovascularization can be observed in the first days after MI and these newly-formed vessels are derived from pre-existing blood vessels in the border zone [1]. This process, referred to as angiogenesis, requires a complex interplay between the so-called tip and stalk cells, which are responsible for the proper formation of new blood vessels. Several signaling pathways, including Notch and WNT signaling, are implicated in angiogenesis [9]. The effect of interventions in WNT signaling on the angiogenesis in the infarcted heart will be discussed in the following paragraphs.

### 8.1. Ligand Level

In two studies targeting the pathway at the level of the WNT ligand production and secretion, an increased neovascularization was observed. Mice overexpressing WNT10b in CMs showed a doubling of the vascular density in the infarct area, with larger diameter blood vessels and more α-smooth muscle actin^+^ smooth muscle cells at 3 weeks after cryoinjury. Moreover, ~30% more ECs could be detected in the infarct by flow cytometry. The WNT10b overexpression activated β-catenin-mediated WNT signaling in the ECs, as demonstrated by immunohistochemistry [35]. A 66% increase in blood vessels near the infarct area was also observed in macrophage-specific Wntless knockout mice, albeit in this study predominantly smaller blood vessels (<20 µm diameter) were detected [12]. Although these results appear to be contradictory at first sight, the differences in approach (specifically targeting WNT10b in CM vs. lowering the secretion of all WNTs in macrophages) make these studies difficult to compare. In contrast, AAV9-mediated overexpression of WNT11 did not affect angiogenesis in the border zone and remote area, as evaluated by CD31 staining [67].

### 8.2. Receptor Level

In several studies, the effects of interventions at the level of the WNT receptors and extracellular scavengers were addressed. In FrzA Tg mice, capillary density was found to be lower in the scar at 7 days post-MI when compared to WT mice, but higher at day 15 post-MI. Moreover, the percentage of muscularized arteries and the vessel surface in the infarct area were significantly increased in the Tg mice at the later time point [44]. EC-specific overexpression of sFRP1 under control of the Tie-2 promoter resulted in a ~50% increase in capillary density, which was not observed in CM-specific transplanted sFRP1 overexpressing bone marrow cells [45]. Along similar lines, injection of sFRP4 in the border zone of the infarcted rat heart resulted in a doubling of the capillary density in the border zone [46].

A threefold increase in blood vessel density was observed in a study from our group, where infarcted mouse hearts were treated with the WNT5a fragment UM206 [41]. Although no direct measurements were performed, UM206 administration to swine undergoing I/R injury resulted in the significant upregulation of Fzd4 and a trend towards increased Vascular Endothelial Growth Factor (VEGF)-expression, both indications of an increased angiogenic response [42]. Injection of the LRP5/6 antagonist DKK2 around the infarct enhanced the neovascularization in models of MI and hind limb ischemia by stimulating angiogenic sprouting of ECs. In contrast, DKK1 was reported to suppress angiogenesis [50]. This observation obscures the mechanism of action of this peptide. Taken together, these studies consistently show a stimulating effect of inhibition of WNT signaling at the extracellular level on angiogenesis.

### 8.3. Destruction Complex Level

The β-catenin destruction complex can be modulated by intervening at different components. So far, studies have been published in which the neovascularization was investigated after cardiac injury in the presence of inhibitors of GSK3β (NP12 and SB415286) or CK1 (pyrvinium). Activation of the WNT/β-catenin pathway by the GSK3β inhibitor NP12 resulted in an increased vascular density at 7 and 14 days post-MI. This was paralleled by increased migration of ECs and sprouting of aortic rings [53]. Supporting evidence for a pro-angiogenic effect of GSK3-inhibition was recently provided by Badimon et al., who showed increased expression of VEGF as early as 1 h after induction of ischemia. However, this VEGF induction was counteracted when the ischemia was followed by 90 min of reperfusion [55]. Saraswati et al. demonstrated that administration of the CK1 inhibitor pyrvinium pamoate into subcutaneous polyvinyl alcohol sponges increased the organization and vascularization of granulation tissue [56]. Pyrvinium administration by oral gavage for 14 days significantly increased the capillary density in the infarct area but not in the border zone [57]. These results are in disagreement with the GSK3-inhibition studies, as GSK3 inhibition activates WNT/β-catenin signaling, whereas CK1 inhibition inhibits this signaling pathway.

### 8.4. Nuclear Level

The effect of direct intervention in nuclear WNT signaling was studied by adenovirus-mediated overexpression of a constitutively active β-catenin mutant in rat MI. At 7 days post-MI, capillary density was increased by ~65% in the Ad-β-catenin group, compared to mock-treated rats. This observation was supported by an increased VEGF-expression in the Ad-β-catenin rats at 7 and 14 days post-MI [59]. In contrast, inhibition of β-catenin/TCF-dependent gene transcription with SEN195 was also reported to increase peri-infarct arteriolar density, in this case by ~42% [15]. These contrasting observations leave undecided whether WNT/β-catenin signaling should be stimulated or inhibited in order to increase the vascular density in the infarct area.

## 9. Effect of Interventions in WNT Signaling on Cardiac Fibrosis

Fibrosis is one of the essential consequences of a MI and a hallmark of the development of HF. CFs and their activated forms, the MFs, are crucial cell types that control the fibrotic progress post-MI and their manipulation–along with strategies aiming to target fibrosis in the infarct and the remote areas–has been repeatedly associated with the WNT signaling pathway [4]. Therefore, fibrosis is assessed in most of the studies included in this systematic review.

### 9.1. Ligand Level

Systemic overexpression of WNT11 via a rAAV9 vector exhibited an anti-fibrotic effect on mice 8 weeks post-MI. The WNT11-mediated effect led to an attenuation of fibrosis in whole-heart sections, in the remote as well as in the perivascular areas, compared to the AAV9-LacZ group (controls) [67]. CM-specific overexpression of WNT10b was shown to confer anti-fibrotic effects following cryoinjury. Indeed, overexpressing WNT10b led to a remarkable suppression in MF numbers (~50% reduction) compared to WT mice, as shown by flow cytometry. Interestingly, a) CF numbers remained the same, implying an effect on transdifferentiation of CFs to MFs rather than an effect on proliferation, and b) the reduction in MF presence correlated well with a very robust decrease in relative fibrotic density of ~60%) [35].

We identified three studies targeting Porcupine in order to manipulate fibrosis post-MI. WNT-974 was shown to suppress ECM deposition following MI, as determined by picrosirius red staining, exhibiting a modest–but significant–suppression of collagen-rich scar area in the WNT-974 treated animals [37]. Treatment of infarcted hearts with the Porcupine inhibitor CGX1321 for 4 weeks led to a remarkable suppression of the fibrotic deposition, both in the infarct and the border zone, compared to controls [38]. GNF-6231 was shown to have an inhibitory effect on the proliferation of αSMA^+^ cells (MF) in the peri-infarct area 3 days post-MI (a ~2.2-fold decrease), suggesting that this compound possesses anti-fibrotic properties [39]. Lastly, Palevski et al. [12] did not observe significant changes in fibrosis conferred by the genetic deletion of Wntless in cardiac macrophages, following permanent LAD ligation.

### 9.2. Receptor Level

Our group suggested the administration of peptide fragments of WNT5a as a novel anti-fibrotic strategy following permanent MI. Indeed, in our initial study we showed that the peptide UM206 could robustly attenuate the collagen deposition in the infarct area at 2 and 5 weeks post-MI. This finding was correlated with a strong increase in MF numbers in the infarct, suggesting that UM206 turns WNT/β-catenin signaling off, which modulates the behavior of MFs and thus fibrosis [41]. Interestingly, the treatment of pigs for 5 weeks following I/R did not exhibit a significant reduction in collagen expression, but in stark contradiction with the original study in mice, it revealed a marked reduction in MF numbers. It was hypothesized that this paradoxical finding relating to MFs might be due to different levels of mechanical stress in the two species [42]. These inconsistent findings, combined with the lack of reproducibility of the mouse results [43], have attenuated the development of WNT peptide fragments as therapeutic agents for MI.

The group of Barandon et al. showed a pro-fibrotic effect of sFRP1 following permanent MI. Indeed, 2 weeks post-MI the collagen density in the infarcted area of sFRP1-overexpressing hearts more than doubled compared to hearts of WT mice. Interestingly, the group also showed a striking effect on cardiac rupture, which is the deadly result of an imbalance between ECM deposition and breakdown: overexpression of sFRP1 markedly decreased cardiac rupture incidence, a phenomenon that was associated with effects on MMP-9 levels [44]. This interesting finding relating to rupture incidence was confirmed in a follow-up study by the same group, where mice transplanted with bone marrow cells from mice overexpressing sFRP1 demonstrate similarly lower rupture risk [45]. Mice with a genetic sFRP2 deletion exhibit an anti-fibrotic phenotype 2 weeks post-MI, as fibrosis is shown to drop to ~15–20% of total LV, compared to ~25–30% in WT littermates. In addition, hydroxyproline (an indicator of deposition of mature collagen deposition) is also found to be markedly suppressed in these sFRP2 KO mice, while in vitro data point towards an inhibitory effect on procollagen C as an explanation [68]. In striking contrast to this study, He et al. demonstrate an opposing result, with sFRP2 injections conferring a strong (~50% suppression) anti-fibrotic effect in rat hearts 2, 3 and 4 weeks post-MI. The authors speculated that the discrepancy with the data reported by Kobayashi et al. might be due to different effects of sFRP2 on BMP1 [69]. In a similar fashion, treatment of mice with sFRP4 post-MI can confer a protective action against the fibrotic scar (termed “acellular fibrous scar” by the authors) development [46].

Three studies were identified on the role of DKK in fibrosis following MI. Injection of DKK1 in mouse hearts led to increased Collagen III mRNA levels in the infarct at 4 weeks post-MI [36]. Local injection of DKK2 in infarcted rat hearts resulted in a remarkable anti-fibrotic effect, with fibrotic deposition per LV area measured being suppressed ~5-fold. Nevertheless, no fibrotic gene expression or MF density was measured, in order to pinpoint the exact ECM components affected by the upregulation of DKK2 in the rat hearts [50]. DKK3 also appears to attenuate interstitial fibrosis when it is genetically overexpressed in mice, as it suppresses total LV collagen volume and mRNA levels of Collagen I and III. In contrast, DKK3 KO mice exhibit a pro-fibrotic phenotype, with substantially increased LV collagen volume and large increases in mRNA levels, especially of Collagen III, compared to the WT littermates. A limitation that we have noted in this study was the lack of data related to the fibrosis specifically in the infarct area and the border zone [51]. At present, the underlying mechanism explaining the variable effects of the different DKK homologues on cardiac fibrosis following MI is unclear.

### 9.3. Destruction Complex Level

Using the model of CM-specific deletion of GSK3β, fibrosis was reported not to be increased in the infarct area compared to WT littermates [52]. On the other hand, the GSK3β allosteric inhibitor NP12 was found to robustly suppress the fibrotic response in the infarct area compared to controls, 7 or 14 days post-I/R [53]. Along similar lines, BIO treatment between day 1 and 14 after MI was shown to cause an attenuation of CF proliferation and to halve the fibrotic deposition in the LV [54]. These results suggest that pharmacological inhibition of GSK3β can confer anti-fibrotic effects in the infarcted heart.

The anti-fibrotic effects of the CK1 inhibitor pyrvinium were investigated in the study of Murakoshi et al. [57]. The group demonstrated that pyrvinium inhibits the proliferation of CFs at day 4 post-MI, robustly suppresses the presence of MFs at day 7 post-MI in the border zone and the infarcted area and attenuates LV fibrosis at day 14 after injury. A limitation identified in this study was that the authors did not measure the effect of their intervention on WNT signaling, hence it is unclear whether the aforementioned inhibitory effects on fibrosis were associated with a clear inhibitory effect on WNT/β-catenin signaling. Unfortunately, in the study by Saraswati et al. using the same compound, fibrosis was not assessed [56].

### 9.4. Nuclear Level

One interesting study has been identified, making an association between β-catenin overexpression and MF activation following MI. Although the authors do not report any in vivo data concerning fibrosis deposition or gene expression of pro- and anti-fibrotic genes, they show that following gene transfer, β-catenin is successfully transfected in MFs and leads to an enhanced expression of αSMA (thus increasing transdifferentiation of CFs to MFs), decreased apoptosis and activated cell cycle in activated MFs, all pointing towards a pro-fibrotic phenotype [59]. In contrast, several studies have focused on the beneficial role of the inhibition of WNT/β-catenin signaling in attenuating fibrosis following ischemia. Paradoxically, Duan et al. showed that conditional deletion of β-catenin in the CFs can also confer a pro-fibrotic effect. The authors underlined the essential role of β-catenin, as they reported dramatically decreased CF numbers when β-catenin was specifically deleted in CFs. This might actually be the reason why in these mice, granulation tissue is disorganized and collagen is barely found within the infarct, 8 days post-I/R [14]. The agent ICG-001-a downstream WNT/β-catenin signaling inhibitor-was not found to mediate fibrosis 7 or 10 days post-MI in rats [61]. Nevertheless, injection of the same agent around the infarct area could lead to the downregulation of S100A4, which attenuated β-catenin levels and reduced αSMA levels following MI in mice. Unfortunately, in this study no further characterization of the infarct area was reported [74].

## 10. Effect of Interventions in WNT Signaling on Cardiac Regeneration

In the last decade, a growing number of studies has addressed the concept of activating a regeneration process in the injured heart to optimally restore its functionality [75]. Traditionally, adult CMs were seen as terminally differentiated cells which are unable to re-enter the cell cycle and proliferate. However, this dogma is now challenged by an increasing number of reports, showing that activation of different signaling pathways can re-activate the cell cycle in adult CMs beyond the point of cytokinesis to produce two functional daughter cells [5]. One of the signaling pathways studied in this context, is WNT signaling [76]. There are several issues compromising the unambiguous detection of adult CM proliferation in in vivo models. First of all, CMs tend to enter the cell cycle, but fail to complete this cycle by cytokinesis, yielding polyploid or multi-nucleated CMs rather than fully divided cells [5]. Because BrdU incorporation merely demonstrates DNA replication, it cannot discriminate between these different processes. Hesse et al. formulated additional recommendations for authentic CM division [77] but these were not followed in most of the studies cited above. Although this may mean that CM proliferation is not formally demonstrated in most of the studies, smaller infarcts and improved cardiac function were reported. This indicates that the increased cell cycle activation in the CMs has a beneficial effect on infarct healing. Another issue is the source of the newly-formed CMs: several cell types are proposed, including cardiac progenitor cells [60], epicardial progenitors [61], adult CMs [63] and even CFs [58]. It has to be noted that the existence of resident stem/progenitor cells has been questioned in a consensus statement on CM regeneration [78]. This makes the source of the newly-formed CMs in the infarct area unclear, although it is tempting to speculate that resident CMs underwent dedifferentiation and proliferation to contribute to this pool of cells [76].

### 10.1. Ligand Level

Several studies have addressed the concept of cardiac regeneration by interventions at the level of WNT. Injection of the WNT/β-catenin-activating WNT3a in the border zone of the infarct reduced the number of BrdU-positive CM in the border zone at 1 week post-MI. The authors attributed this effect to a limited renewal of the adult cardiac side population cells [34]. In contrast, CM-specific overexpression of WNT10b, which also is an activator of WNT/β-catenin signaling, was found to induce patches of α-actinin-positive CMs with developing sarcomeric structures [35].

Inhibition of the essential WNT lipidation with the porcupine inhibitor CGX1321 increased the number of EdU-positive CMs in tissue sections of the infarct area. This result was confirmed in CMs, isolated from the infarcted hearts, demonstrating that significantly more new, mononucleated CMs were formed in the CGX1321-treated group at 4 weeks post-MI [38]. A 3-fold increase in TnT^+^/Ph3^+^ CMs was also observed in hearts of WNT-974 treated mice, despite of the fact that these hearts were not injured [37]. Treatment with the porcupine inhibitor GNF-6231 was shown to increase the number of GATA4^+^ cells in the border zone of the infarct. Because this transcription factor is typically expressed by early differentiation CMs, this suggests the induction of CM proliferation by this treatment, which was confirmed by in vitro studies [39].

### 10.2. Receptor Level

Only a single report on the effects of extracellular WNT inhibition was found to discuss cardiac regeneration. Injections of sFRP2 in the infarct border zone enhanced and sustained the expression of CM-specific genes in resident c-Kit^+^ cells in the infarct area, which was triggered by MI [79].

### 10.3. Destruction Complex Level

In several studies, the effects of interventions in the β-catenin destruction complex were studied. CM-specific deletion of GSK3β was reported to increase the number of BrdU^+^/TnI^+^ cells almost 3-fold at 4 weeks post-MI [52]. This observation was confirmed by Kim et al., who showed a more than 2-fold increase in BrdU^+^ CMs in a zebrafish model of CM intoxication with aristolochic acid, treated with the GSK3β inhibitor BIO [54]. These findings suggest that activation of WNT/β-catenin signaling can induce CM proliferation. Similar results were observed in the mouse MI model, where a single injection of the CK1-inhibitor pyrvinium increased the number of Ki67^+^ cells in the peri-infarct and distal myocardium [56]. Interestingly, evidence for in situ reprogramming of CFs into CMs was presented in a mouse MI model treated with a combination of the transcription factors Gata4, Mef2c and Tbx5, together with the TGFβ-inhibitor SB431542 and the axin stabilizing compound XAV939 [58]. It is unclear whether this combination of interventions activates the same molecular mechanisms as the single inhibition of GSK3β activity. It has to be noted, however, that interventions in the destruction complex, and particularly in GSK3β, may have additional effects on signaling pathways other than WNT signaling [80].

### 10.4. Nuclear Level

Interventions at the level of nuclear WNT/β-catenin signaling have also been reported to confer effects on CM proliferation (Figure 2). Using a mouse model of CM-specific β-catenin depletion, Zelarayán et al. observed a layer of small cardiac TnT^+^ and Sca1^+^ cells in the subepicardial and subendocardial borders of the infarct area at 4 weeks post-MI, which was less prominent in the WT controls [60]. Similar observations were reported in a rat MI model, where subcutaneous administration of the WNT/β-catenin inhibitor ICG-001 resulted in more CMs with BrdU-positive nuclei near the epicardium at 7 days post-MI [61]. Using zebrafish embryo-based screening techniques, Xie et al. identified two related compounds, Cardiomogen-1 and -2, which induce CM hyperplasia by inhibiting WNT/β-catenin signaling. These compounds were tested in a zebrafish model of apex amputation and in a mouse MI model and stimulated the formation of newly formed CMs in both models [63]. These studies consistently demonstrate that inhibition of WNT/β-catenin signaling at the nuclear level can augment the proliferation of CMs around the infarct.

## 11. Concluding Remarks

The effect of interventions in the WNT signaling pathway on infarct healing has been subject of study for nearly two decades. It is quite common for such a new research area that the initial studies show variable–and sometimes even conflicting–results. This has motivated us to perform a systematic analysis of studies describing interventions in the pathway in in vivo models of cardiac injury, in order to identify common findings or trends. In total, we identified 32 studies via literature search and added 6 papers from our own archives fitting the inclusion criteria. We analyzed them for data on infarct size, cardiac function, apoptosis, fibrosis and angiogenesis. Moreover, we put specific emphasis on any reported signs of cardiac regeneration, as WNT signaling is increasingly associated with this interesting phenomenon.

It is relevant to note that in 29 of these 38 studies, the intervention was aimed at inhibiting WNT signaling, whereas in 4 there was a direct stimulation of the pathway by increasing WNT protein of β-catenin expression. In the remaining studies, an inhibitor of the pathway (mainly GSK3β) was inactivated, leading to an indirect stimulation. The vast majority of the studies (>80%) showed beneficial effects on one or more of the parameters that we included in our analysis. Taken together, this demonstrates that inhibition of WNT signaling at different levels of the pathway is beneficial for infarct healing. The major deviation from this pattern was found in interventions in GSK3β that were reported to be beneficial, despite the fact that GSK3β inhibition activates the signaling pathway.

A major problem that we encountered was that the WNT signal transduction pathway is highly complex, with multiple family members at different levels (19 WNT protein subtypes, 10 FZDs, 5 sFRPs, and 4 DKK protein subtypes). This has complicated our systematic review, because in many cases only a single report on the targeting of an individual pathway member was found. Fortunately, there are exceptions in which drugs where used that have a more general mode of action. In three studies, inhibitors of porcupine were used which prevent the biological activation of virtually all WNT proteins and all of these studies showed beneficial effects on infarct healing. Along similar lines, in 5 studies the interaction of β-catenin with its transcription complex was targeted and 4 out of these studies showed beneficial effects on infarct healing. On the other hand, in 4 studies it was shown that inhibition of GSK3β with either a drug or a genetic intervention improved infarct healing. Because GSK3β targets β-catenin for degradation, GSK3β inhibition is generally considered to activate WNT signaling. However, it has to be noted that this kinase not exclusively serves in WNT signaling but is actually involved in a plethora of other signaling pathways, questioning the specificity of these interventions for WNT signaling. Along similar lines, WNT/β-catenin signaling does not operate in isolation in the nucleus but may crosstalk with other signaling pathways such as TGFβ and YAP/TAZ signaling [76]. Therefore, interventions at this level may also affect other signaling pathways. Furthermore, β-catenin is an essential component of the cell adhesion complex, which may add to the effects of interventions in WNT signaling on infarct healing.

In this systematic review, we tried to identify the mechanism(s) that were modulated by the interventions in order to improve the infarct healing. Therefore, we systematically analyzed the studies for results on apoptosis, angiogenesis, inflammation and fibrosis, which all have been described to be regulated by WNT signaling. This analysis turned out to be quite challenging: although in most studies effects on infarct size and cardiac function were reported, there was little uniformity in the evaluation of the underlying responses to injury. Therefore, we cannot draw firm conclusions regarding a single causal mechanism in infarct healing that can be augmented by inhibiting WNT signaling. In this context, it is interesting to mention that, particularly in the more recent publications, increased cardiac regeneration has been mentioned as a driving force behind the beneficial effects of the inhibition of WNT signaling on infarct healing. This fits nicely with the observations in zebrafish and newts that endogenous inhibition of WNT signaling is a prerequisite for the cardiac regeneration that occurs spontaneously in these species upon cardiac injury [81].

Based on our systematic analysis of these 38 publications, we provide the following recommendations for future studies:

1. a broad targeting of WNT signaling, preferably with a drug/therapeutic agent, appears to be more informative than an intervention aiming at a single member of the extensive family
2. a comprehensive analysis of the wound healing response is recommended, including all the aspects of wound healing that we analyzed in this systematic review
3. specific focus should be on signs of cardiac regeneration and CM proliferation, because this would be of major interest for translation towards the clinic.

## Figures and Tables

**Table 1:** Overview of the main results of the studies included in this systematic review. Abbreviations: AAV9: Adeno associated virus serotype 9; Ad: adenovirus; α-MHC: myosin heavy chain-α; βcat: β-catenin; BMC: bone marrow cell; BZ: border zone; CF: cardiac fibroblast; DKK: Dickkopf; FZD: frizzled; GSK3: glycogen synthase kinase-3; i.p.: intraperitoneal; I/R: ischemia/reperfusion; KO: knockout; LRP: LDL receptor-related protein; MI: myocardial infarction, OE: overexpression; s.c.: subcutaneous; sFRP: secreted frizzled-related protein; Tg: transgene; VEGF: vascular endothelial growth factor; WIF: WNT inhibitory factor; ↑: increase, ↑: decrease; →: differentiation; ↔: no change, - : not determined.

	Intervention/Model/Follow Up	Infarct Size	Cardiac Function	Cardiomyocyte Apoptosis	Inflammation	Angiogenesis	Fibrosis	Cardiac Regeneration
**Ligand**								
Palevski et al. 2017 [12]	Mouse MI, macrophage-specific Wntless KO (30 d)	-	↑	-	M1→M2	↑	↔	-
Oikonomopoulos et al., 2011 [34]	Mouse MI, rWNT3a injection in BZ (7d)	↑	↓	-	-	-	-	↓
Paik et al., 2015 [35]	Mouse MI, α-MHC-WNT10B Tg (3 w)	↓	↑	-	↓	↑	↓	yes
Moon et al., 2017 [37]	Mouse MI, WNT-974 (10 w)	↓	↑	-	-	-	↓	yes
Yang et al., 2017 [38]	Mouse MI, CGX1321 (4 w)	↓	↑	↔	-	-	↓	yes
Bastakoty et al., 2016 [39]	Mouse MI, GNF-6231 (1 w)	↓	↑	↓	-	-	↓	yes
Morishita et al., 2016 [67]	Mouse MI, AAV9-WNT11 (8 w)	-	↑	↔	↓	↔	↓	-
**Receptor**								
Wo et al., 2016 [36]	Mouse MI, DKK1 and IGFBP4 Injection (2-4 w)	↓ (IGFBP4)	↑ (IGFBP4)	-	-	-	Col-III ↑ (DKK1)	-
Fan et al., 2018 [40]	Mouse MI, immunized with FZD1 (8 d)	↔	↑	↔	-	-	-	-
Laeremans et al., 2011 [41]	Mouse MI, UM206 s.c. (5 w)	↓	↑	-	-	↑	↓	-
Uitterdijk et al., 2016 [42]	Swine I/R, UM206 s.c. (5 w)	↓	↔	-	-	↑	↔	-
Daskalopoulos et al., 2019 [43]	Mouse MI, UM206 s.c. (3–8 w)	↔	↔	-	-	-	↔	-
Barandon et al., 2003 [44]	Mouse MI, FrzA Tg (30 d)	↓	↑	↓	↓	↑	↑	-
Barandon et al., 2011 [45]	Mouse cryoinjury, sFRP1 Tg BMCs (15 d)	↓	↑	-	↓	↔	-	-
Matsushima et al., 2010 [46]	Rat MI, sFRP4 injection in BZ, (20 w)	↓	↑	-	-	↑	↓	-
Nakamura et al., 2016 [47]	Mouse I/R, sFRP5 KO (24 h)	↑	↓	↑	↑	-	-	-
Meyer et al., 2017 [48]	Mouse MI, WIF1 KO and AAV9-OE (4 w)	KO↑, OE↓	KO↓, OE↑	-	KO↑, OE↓	-	-	-
Borrell-Pages et al., 2016 [49]	Mouse MI, LRP5 KO (60 min)	↑	-	-	-	-	-	-
Min et al., 2011 [50]	Rat MI, DKK2 injection in BZ (1 w)	↓	↑	↓	-	↑	↓	-
Bao et al., 2015 [51]	Mouse MI, DKK3 KO; α-MHC-driven DKK3 Tg (1 w)	KO↑, Tg↓	KO↓, Tg↑	KO↑, Tg↓	KO↑, Tg↓	-	KO↑, Tg↓	-
Kobayashi et al., 2009 [68]	Mouse MI, sFRP2 KO (15 d)	-	↑	-	-	-	↓	-
He et al., 2010 [69]	Rat MI, sFRP2 injection in BZ (4 w)	↔	↔	↔	-	-	↓	-
Hodgkinson et al., 2018 [79]	Mouse MI, sFRP2 injection in BZ (2 d)	-	-	-	-	-	-	yes
**Destruction complex**								
Woulfe et al., 2010 [52]	Mouse I/R, CM-specific GSK3β KO (8 w)	↔	↑	↓	-	-	↔	yes
Baruah et al., 2017 [53]	Mouse I/R, GSK3-inhibitor NP12 (2 w)	↓	↑	-	-	↑	↓	-
Kim et al., 2016 [54]	Rat MI, GSK3 inhibitor BIO i.p. (2 w)	↓	↑	-	M1→M2	-	↓	yes
Badimon et al., 2019 [55]	Mouse I/R, GSK3 inhibitor SB415286 (90 min)	↓	-	-	-	VEGF↑	-	-
Saraswati et al., 2010 [56]	Mouse MI, pyrvinium injection in BZ (30 d)	↔	↔	↔	-	↑	-	yes
Murakoshi et al., 2013 [57]	Mouse MI, pyrvinium oral gavage (2 w)	↔	↑	-	-	↑	↓	-
Mohamed et al., 2017 [58]	Mouse MI, GMT cocktail+TGFβi +XAV939 (12 w)	↓	↑	-	-	-	-	yes
**Nuclear**								
Duan et al., 2012 [14]	Mouse I/R, epicard/CF specific βcat KO (8 d)	↔	↓	-	-	-	↓	-
Matteucci et al., 2016 [15]	Rat MI, SEN195 s.c. (4 w)	↓	↑	-	-	↑	-	-
Hahn et al., 2006 [59]	Rat MI, Ad-βcat OE (1 w)	↓	↑	↓	-	↑	-	-
Zelarayán et al., 2008 [60]	Mouse MI, CM-specific βcat KO (4 w)	↓	↑	↔	-	-	-	yes
Sasaki et al., 2013 [61]	Rat MI, ICG001 s.c. (4 w)	↔	↑	-	-	-	↔	yes
Sun et al., 2019 [62]	Rat MI, ICG001 i.p. (3 w)	↓	↑	↓	-	-	-	-
Xie et al., 2019 [63]	Mouse MI, cardiomogen i.p. (3 w)	↓	↑	-	-	-	-	yes
Qian et al., 2018 [74]	Mouse MI, ICG001 injection in BZ (1 w)	-	-	-	-	-	↓	-
